# Becoming a teacher can reduce obedience compared to being solely an examiner. Agentic state and obedience in the Milgram paradigm

**DOI:** 10.3389/fpsyg.2025.1613507

**Published:** 2025-10-15

**Authors:** Tomasz Grzyb, Dariusz Dolinski

**Affiliations:** Faculty of Psychology in Wroclaw, SWPS University, Wroclaw, Poland

**Keywords:** obedience, Milgram paradigm, teaching vs. examining, social influence, compliance

## Abstract

Studies of obedience carried out in the Milgram paradigm tend to report shockingly high levels of obedience from people who are ordered by an authority figure to eventually, if administer all required shocks, electrocute another person. In the psychology literature, the person who carries out these commands is called the teacher. The authors of the present article note, however, that the term “examiner” would be more appropriate here, since the study participant is limited to verifying the correctness of the responses given by the student, i.e., the person sitting behind the wall. It was assumed that if the participant actually performed the role of a teacher (and thus first taught the “student,” and only then checked the correctness of the answers to questions), the level of obedience demonstrated would be reduced. The results of our experiment partially confirmed this assumption. In the examiner condition, 4 out of 40 participants (10%) refused to press all ten switches, meaning that 90% proceeded to 150 V. In the conditions where participants had first taught the student, refusals occurred more than twice as often: 9 out of 40 (22.5%), with 77.5% reaching 150 V. This difference, however, was not statistically significant. We also analyzed an indirect measure of non-compliance—the frequency with which the experimenter had to prompt participants to continue by reciting the standardized phrases prescribed by the procedure whenever participants expressed hesitation or refused to comply. These experimenter interventions were more frequent in the conditions where participants had previously taught the learner (Median = 0.5) than in the examiner condition, where their role was limited to punishing learner for his mistakes (Median = 0). This difference was statistically significant.

## Introduction

The studies of Milgram (1963, 1965), in which participants were convinced that they were engaged in an experiment on the role of punishment in the learning process, are classics of psychological research (see Blass, 2000, 2004; Miller, 2004; Perlstadt, 2013). Milgram revealed that under certain conditions a clear majority of people would obey a succession of commands to shock a person sitting behind a wall with electricity of increasing voltage, ultimately reaching 450 volts if instructed to do so by the professor conducting the study. Certainly, it should be noted that Milgram conducted 24 different experimental conditions, in which only a part resulted in a clear majority (two-thirds) administering all shocks.

The numerous explanations for such remarkable obedience train their focus on very different aspects. Miller et al. (1995), for example, point to the social norms that function in society according to which we should not suddenly and overtly defy authority figures. If we do, we will be judged badly by our peers and disliked. Aware of this, participants avoid such conduct and obediently push more switches on the electric shock generator. Lutsky (1995) focuses on the element of total surprise that participants face. They arrive at the laboratory believing that their learning and memory abilities will be tested, and they are then assigned an entirely different role. Gilbert (1981), in turn, focuses on the human tendency to continue an activity that has been initiated. The participant is initially instructed to press the first switch of the electric shock generator, a de facto way of sending the student a tactile message that a mistake has been made, rather than causing any pain. Individuals who carry out this instruction will also comply with the next command (because this shock is only minimally stronger), and if they do, they will fulfill the next command, and so on and so forth (Dolinski and Grzyb, 2016; Grzyb and Dolinski, 2021).

A promising reinterpretation of Milgram's findings frames many completions as driven less by “blind obedience” and more by a fear of face-to-face confrontation with the experimenter (Russell and Künstler, 2024). Milgram himself noted a “competing inhibition” that made subjects dread appearing “arrogant, untoward, and rude” if they broke off “to his face,” pointing to the social cost of open defiance as a motivational barrier to disobedience. Corroborating this, conditions in which stopping did not require initiating a direct clash with the experimenter produced very low completion (mean = 11.25%), whereas conditions that did require such a clash yielded much higher completion (mean = 54.61%), a pattern that is statistically significant (Künstler, 2024).

On the other hand, (Reicher and Haslam, 2011; Reicher et al., 2012; Haslam et al., 2015; Haslam and Reicher, 2017) suggest that the participant's social identification with science (defined as perceived membership in a group, in this instance as a “good” participant or subject) and its values is crucial, and that the experimenter embodies those very values. The researchers posit that individuals examined within Milgram's paradigm are active followers rather than those who obediently adhere to the experimenter's instructions against their own values and moral compass. However, this assumption starkly contradicts both the accounts of participants' behavior in this paradigm (Milgram, 1974; Dolinski and Grzyb, 2020) and the findings of recent research conducted within the Virtual Reality (VR) paradigm. The latter reveals that participants undergo considerable stress and emotional tension in such scenarios, challenging the notion of unquestioning compliance (e.g., Caspar et al., 2020, 2022; Cheetham et al., 2009; Dambrun et al., 2014; Slater et al., 2006; Götz et al., 2023). Lepage et al. (2018) conducted an experiment monitoring heart rate variability (a biomarker of stress vulnerability). They demonstrated that higher resting HRV reduced destructive obedience. It is reasonable to posit that whether stress arises from personal distress or empathy, in both scenarios, it inclines the subject toward refusing to continue pressing the buttons for the electric shock generator. This refusal serves as a mechanism to alleviate the aversive feeling of stress.

The participants in Milgram's experiments not only acted as engaged followers but also displayed uncertainty in determining their behavior, as indicated by the necessity for the experimenter to employ prods outlined in the experimental design. This uncertainty is further underscored by the experimenter resorting to additional arguments beyond the designated prods to ensure participant compliance, emphasizing the inadequacy of the prescribed prompts for this purpose (e.g., Perry, 2013; Hollander, 2015). These observations starkly challenge the fundamental assumption of Reicher and Haslam's (2011) model. In addition, a detailed analysis of the post-experimental interviews conducted with Milgram's participants has shown that the assumption that they obeyed solely because they identified with the experimenter is a far-reaching oversimplification. In fact, they provided a wide variety of explanations for their behavior in the laboratory (Hollander and Turowetz, 2017, 2018). It should also be noted that explaining their reactions after the fact in terms of contributing to science and identifying with the experimenter's objectives may have served merely as a strategy to protect their self-esteem.

Furthermore, contrary to the claims of its proponents, empirical support for the concept of engaged followership is very weak. In one study (Reicher et al., 2012), participants were presented with scenarios from Milgram's experiments and asked to estimate the degree to which the participant in each case identified with (a) the experimenter and scientific values, and (b) the learner and the broader community he represented (According to the theory, the latter identification should weaken participants' willingness to punish the learner). The authors reported that obedience correlated positively with identification with the experimenter and scientific values, while refusal to obey correlated with identification with the learner and the community.

However, they failed to acknowledge that their own findings actually contradict their theory. In Milgram's most widely cited experiment—the so-called *new baseline condition* (Experiment 5; Milgram, 1974)—experts in social psychology estimated that identification with the learner was higher than identification with the experimenter. According to Haslam and Reicher's framework, such conditions should have led to particularly frequent refusal to obey. In reality, the opposite occurred: this experiment produced one of the highest rates of full obedience (62.5%).

Another study cited as evidence for the engaged followership account (Haslam et al., 2014) is in fact unrelated to Milgram's paradigm, despite the authors' claims to the contrary. In that study, participants faced no moral dilemma, inflicted no harm, and experienced no genuine pressure from an authority figure; moreover, the procedure was conducted entirely online.

Finally, recent research by Grzyb et al. (2025) further undermines the engaged followership model. They demonstrated that when the alleged “word memorization” study was framed as a purely commercial marketing test, obedience levels were just as high as when participants believed the study served strictly scientific purposes.

Social psychologists delved also into personality factors that could potentially influence subjects' inclination to follow the experimenter's instructions. Factors taken into consideration included traits such as extroversion-introversion, interpersonal trust, social intelligence, locus of control (Blass, 1991), religiousness (Bock and Warren, 1972), empathy (Burger, 2009; Dolinski et al., 2017), neuroticism (Zeigler-Hill et al., 2013), need for cognitive closure (Grzyb et al., 2018), authoritarianism (Elms, 1972; Caspar, 2011; Caspar et al., 2021), and conscientiousness and agreeableness (Bègue et al., 2015).

Nevertheless, the original interpretation advanced by Milgram himself (1974) remains relevant. Milgram notes that in various social situations, people can function in one of two modes. Individuals can act autonomously, consciously directing their behavior, or they can enter an agentic state, where they carry out orders of an authority figure. In several of his conditions, Milgram assumes that participants in his experiments did not feel like agents of their own actions. On the contrary, they assumed roles of subordination to the experimenter and did not feel full responsibility for their own actions. Although they experienced acute stress and tension, resulting from the awareness that they were doing serious harm to another human being, they were unable to withdraw from the situation and refuse their further participation in the experiment. Certain data collected by Milgram (1974), and particularly by Kilham and Mann (1974), point to the correctness of this interpretation.

On the other hand, certain findings seem to challenge the explanation of participants' obedience solely in terms of the agentic state. One such line of evidence comes from Milgram's own research on the perceived distribution of responsibility (Blass, 1996). After completing the study, participants were asked to assign percentages of responsibility for the learner's suffering to three parties: the learner himself (for making mistakes), the participant (i.e., teacher), and the experimenter. The total had to equal 100%. According to the concept of the agentic state, participants should have placed primary responsibility on the experimenter. In fact, the results were more nuanced: participants who disobeyed saw themselves as mainly responsible, while those who were fully obedient attributed only slightly more responsibility to the experimenter than to themselves.

It is important, however, to interpret these findings with caution. This was a *post hoc* assessment, carried out after participants had already complied with the experimenter's instructions and administered shocks. In such circumstances, self-reflection and self-blame are likely. Their perception of the experimenter's responsibility in the *moment of action*—sitting in front of the shock generator and hearing the prods—may have been quite different.

Another frequently cited objection to the agentic state interpretation concerns the nature of the experimenter's prods (Gibson, 2013). Of the four standardized prompts, the first three were phrased as requests or suggestions rather than orders, while only the fourth was an unequivocal command. Critics argue (e.g., Reicher and Haslam, 2011) that if participants were truly in an agentic state—perceiving themselves as mere instruments of the experimenter's will—then polite requests should not have sufficed; only a clear order should have been effective. Yet the opposite was observed: the first three prods were generally successful in persuading participants to continue, whereas the fourth always led to outright refusal.

We believe this issue is more complex than such critiques suggest. First, even a politely phrased request from an authority figure can be perceived by a subordinate as an implicit command. From this perspective, it is unsurprising that participants in an agentic state complied with these apparently “mild” signals. Second, prod 4 was problematic not only because it was an order, but also because it violated the explicit contract stated at the beginning of the experiment: participants had been assured that they could withdraw at any time. Hearing that they had “no choice” directly contradicted this promise, prompting many to rebel and terminate their participation.

For these reasons, we do not find convincing empirical grounds to reject the agentic state explanation of obedience. On the contrary, we maintain that many participants were indeed operating within such a state. Moreover, we suggest that the agentic state in Milgram's paradigm involved not only submission to authority, but also the perception that participants bore no responsibility for the learner's errors, which were presented as entirely the learner's own doing.

Note that in the reality of the school, the teacher first teaches the student, and then tests that student's level of knowledge. The student's good result is also the teacher's success (being partially responsible for it), while a bad result also reflects negatively on the teacher. This is because of a failure to adequately prepare the student for the exam, or to motivate the student to prepare independently at home. Not surprisingly, then, teachers in real life situations, feel and attribute to themselves a portion of the responsibility for both the successes and failures of their students (e.g. Guskey, 1981; Daniels et al., 2018). With regard to Milgram's experiment, the participant is referred to in the psychological literature as “teacher” (the term also used by Milgram), while in fact a more accurate label would be “examiner”, since the role of the participant is reduced solely to checking whether the student's responses are correct (in other words: whether a sufficient level of independent learning has been reached) and punishing the student for mistakes. It is important to highlight a significant difference between the roles of examiner and teacher. The examiner is not in a position to assess whether a sufficient level of independent learning has been achieved. Only the teacher has the capacity to make such a judgment. The participant, acting as the “good subject,” would only be able to punish the student for wrong answers after the four prompts fail to elicit obedience to administer the next higher shock.

However, we may pose the question of what would happen if, at the beginning of the experiment, the participant actually performed the role of a teacher and genuinely taught the student. With respect to mistakes made by the student in the second phase, the participant should then feel at least somewhat responsible for these failures. Assuming the following definition of responsibility, “a sense of internal obligation and commitment to produce or prevent designated outcomes, or that these outcomes should have been produced or prevented” (Lauermann and Karabenick, 2011, p. 135), it can be assumed that in such a situation participants should be more in an autonomous state than those in a classic Milgram experiment in which the “learner” is behind the wall in a separate room. This, in turn, should reduce the participant's tendency to obey authority, manifested in a diminished tendency to obey the experimenter's commands and administer an electric shock to the student.

An additional justification for this hypothesis is the possible change in the perception of the “student” by the “teacher”, which proceeds from membership in the “WE” category, that is socially identifies as the “good” subject.

In several experimental conditions devised by Stanley Milgram, it remains ambiguous as to which individual, be it the experimenter or the learner, the participant perceives as psychologically closer. Consequently, the identification of the participant with either figure remains uncertain.

On the one hand, both the teacher and the student are participants recruited through an announcement, who come to the experimenter (and therefore, at least in theory, constitute a common group). On the other hand, however, after a short period of time, the teacher and experimenter remain in the same room and de facto cooperate with each other (the experimenter instructs the teacher and provides information about the study), while the student remains out of sight, behind a wall. Introducing conditions in which there is actually real cooperation between the teacher and the student in learning the material can create a bond between them and a sense of belonging to the category of “WE.” The affiliation within a shared collective ‘WE' category fosters a sense of non-harm toward one another, and at times, even prompts actions against those outside this category (e.g., Cialdini, 2016, 2021; Bolt et al., 2016; Loehr, 2022; Jenkins et al., 2021).

Taken together, our analyses suggest that obedience in Milgram's paradigm is shaped not only by submission to authority, but also by how participants construe their own role in the situation. In summary, we argue that Milgram's use of the term “*teacher”* to describe the participant—and its subsequent uncritical adoption in the literature on “obedience to authority”—is misleading. The participant's role in Milgram's paradigm was not that of a teacher, but rather that of an examiner, assessing the performance of another (supposed) participant. Moreover, the very fact of being an examiner, rather than a teacher, may itself contribute to heightened obedience.

In our study, we therefore set out to compare obedience in Milgram's classic paradigm with conditions in which participants assumed the role of an actual teacher—first instructing the learner, and only then testing his performance. We hypothesize that participants placed in the role of a teacher, as opposed to an examiner, will be less inclined to comply with orders to administer increasingly severe electric shocks. Additionally, we anticipated that convincing individuals in the role of teachers to adhere to instructions for administering electric shocks to the learner would prove more challenging than for those in the examiner role. As a result, we conjectured that experimenters would need to prompt teachers more frequently to ensure compliance with the given instructions.

## Method

### Participants

Participants were recruited to take part in the experiment in a manner similar to the original Milgram study—through an advertisement. It was posted on a popular online portal used by people seeking supplemental employment. The ad stated that candidates were being sought to take part in a scientific study on memory, provided a contact telephone number and specified the payment (PLN 100—just over $20). Those willing to take part in the study called the number, and then underwent a verification procedure (students and graduates of social sciences majors were excluded, as well as those betraying knowledge about psychological experiments). After successfully passing the procedure, their visit to the laboratory was then arranged.

Simmons et al. (2011) recommend a minimum of 20 participants per condition in psychological experiments, while also emphasizing that larger sample sizes help guard against questionable research practices. When planning our experiment, we decided to double this number, assuming that 40 participants (20 women and 20 men) would be examined in each condition. One of the participants stated during the experiment that she had heard of a similar study. An additional person thus assumed her place. The average age of participants was 28.62 years (*SD* = 5.51). Independent-samples *t*-tests were conducted to examine potential age differences between groups. No significant difference was found between men and women, *t* = 0.36, *p* = 0.718. Similarly, the comparison between participants assigned to the teacher and examiner conditions revealed no significant age difference, *t* = −1.14, *p* = 0.259.

### Procedure

An extremely important consideration in planning research into obedience based on the Milgram paradigm is to take into account the ethical concerns that have been articulated against the classic procedure. While implementation in its original form is fortunately not feasible today for ethical reasons, relying on the “obedience light” procedure modified by Burger (2009), while maintaining full control over the course of the experiment and providing support from clinical psychologists, is accepted by Institutional Review Boards in many places around the world. We therefore decided to utilize the “obedience light” procedure (and thus the procedure shortened to 10 “electric shocks” instead of 30, as in the original), additionally removing the last cry of the “student” demanding to be released from the lab [While Burger employed a condensed version of Milgram's (1974) study No. 5, our experiment is grounded on Milgram's study 2, a choice made to mitigate ethical concerns].

Other steps we took to minimize stress for study participants were:

- careful selection of participants with questions about wellbeing, physical and mental ailments, with the aim of eliminating those who are exceptionally bad at handling stress,
- informing participants at least three times (including in writing) that they can withdraw at any time without having to return their compensation,
- reducing the duration of the experiment compared to the original Milgram study,
- upon completion of the experiment, immediately informing participants of its true nature (and showing the “student” whole and healthy),
- providing psychological support after the study (by providing the phone number of a clinical psychologist on duty and informing participants that help is available if needed).

The entire procedure was based on version 2 of the Milgram experiment (Milgram, 1974) with changes proposed by Burger (2009). It is worth noting that the key change proposed by Burger was to shorten the duration of the entire study and reduce the stress experienced by participants. Burger achieved this by ending the entire procedure after participants pressed the 10th button (instead of the 30th, as in Milgram's original studies). Naturally, if participants wanted to withdraw earlier, before the 10th button, they had that option.

Upon arriving at the lab, the participants were greeted by a man about 50 years old who introduced himself as the psychology professor conducting the study (in reality he was an actor cooperating with the researchers and playing a set role). They also met a second study participant (actually a confederate)—a man about 30 years old. Both of them (the actual study participant and the confederate) were led by the “psychology professor” to a room where they were informed of the purpose of the study (to test memory performance under punishment conditions), asked to sign a declaration of informed consent to take part in the experiment, and given their gratification. They were also told that the experiment would be carried out in pairs, and that the roles of teacher student in the study would be decided at random. The participants were informed that they could withdraw from the study at any time (the same information was included in the informed consent form they signed).

Next, a rigged random drawing was conducted, in which the real study participant always “drew” the role of the teacher, and the confederate took the part of the student. The experimenter then explained that the student's role would be to learn by heart the associations between certain syllables. Upon hearing the syllable “BA”, for example, he is supposed to respond “BO”, and upon hearing “DA”, he is supposed to respond “DE”. In the “participant-examiner” condition (i.e., the classic conditions of Milgram's studies), the experimenter would hand a piece of paper with eight pairs of syllables to the “student” and tell him that he had a few minutes to learn these associations, then lead him to another room and leave him there. After 8 min, the student would report that he had completed the task.

In the “participant-teacher” condition, the experimenter handed an identical card to the teacher and explained that the task was to teach the student to respond with the second syllable from the pair after hearing the first syllable. Together, the teacher and the student would move to another room and begin learning. The student consistently made fewer and fewer mistakes, and after about 8 min of this training, he was already answering quickly and without errors. The teacher and the student would then jointly report back to the experimenter on the completion of the task.

The second part of the procedure was the same in both conditions. The student stayed in one room, while the teacher and the experimenter went into an adjacent room. The experimenter now explained to the teacher that the study's aim was to test the role of punishment in the learning process, and explained that an electric shock generator was used to apply punishment. In doing so, he showed a device resting on a table, with 30 switches in a row. Each switch had a label indicating the voltage. The first one read 15 volts, the second: 30, the third: 45, and so on until the final one: 450 volts.

The experimenter discussed the operation of the device, showing the participant that pressing individual switches would trigger pulses ranging from 15 to 450 V. He also read captions explaining how painful or harmful each pulse could be. The information on the device was written in English (exactly the same as in the original Milgram experiments), but the experimenter read it to the participant in Polish. At the first switches he said that pressing them would entail a “light shock,” then “moderate shock,” “strong shock,” “very strong shock,” “intense shock,” “extreme intense shock,” “danger-severe shock.” The last two switches were marked only with the symbols xxx, about which the experimenter remarked: “Here they don't even bother using words.” To demonstrate the device's operation, he also attached electrodes to the participant's wrist and pressed the third switch, hitting him with a 45-volt current (pilot studies revealed that the vast majority of people hit with a current of this voltage described their sensations as clearly aversive, but not painful).

The experimenter would then show the participant that another cable coming out of the generator went through the wall into an adjacent room, where the “student” was already sitting. He would then walk into that room with the participant and, in the participant's presence, attach the electrodes to the “student's” wrist. He and the participant would then return to the room where the generator stood. An intercom system facilitated two-way verbal communication between the two laboratory rooms where the study was being carried out. The experimenter asked the participant to sit by the generator and handed them a list of 45 pairs of syllables, written in a different order than on the sheet the student was given. He explained to the participant that the task was to read one of the syllables and wait for the student's answer. Following a correct response, the instruction was to proceed to reading the next syllable. In the case of an incorrect response, the participant was to wait for instruction in the form of the four prods from the experimenter. This would indicate the punishment to be administered to the student, consisting of an electric shock. The experimenter would sit at a distance of about 3 m from the participant, in order to conduct his observations.

The experimenter then instructed the participant to read the first syllable. The student answered correctly, and the participant then read the next syllable. The student made no mistake on this or subsequent attempts, but after hearing the seventh syllable, he erred. The experimenter then instructed the “teacher/examiner” to press the first switch. The “student” from behind the wall responded with a soft grunt. The experimenter would then give the command to read the next syllable. The “student's” response was correct this time. However, at syllable number ten he made another mistake. The experimenter instructed the “teacher/examiner” to press the number 2 switch, after which the “student” responded with a slightly more pronounced grunt. Another mistake followed at syllable number 13. Here, too, the reaction of the “student” to pressing the switch (the third) was a grunt, albeit a slightly stronger one and suggesting that the electrical shock had caused him discomfort. Subsequent jolts of electric current (transmitted at successive errors, which occurred at questions 15, 18, 22, 24, 27, 31, and 34) were accompanied by cries of pain. These cries increased in volume and intensity.

If the “teacher/examiner” hesitated or expressed doubts about using electricity against the “student,” the experimenter urged him or her on, reciting in turn the same prods as in Milgram's studies: “Please continue”, “The experiment requires that you continue”, “It is absolutely essential that you continue”, “You have no other choice, you must go on”. If, after the fourth urging, the participant still refused, the experiment was terminated.

After the study concluded, a very careful debriefing was conducted. First, it was made sure that the participants were convinced that they were indeed electrocuting the person sitting behind the wall (no participant doubted that this was the case); next, the details of the experimental procedure were explained, and an apology was given to the participants for misleading them. The participants were informed that in this type of study, most people would go so far as to push the last switch, but at the same time they were told that this experience could be a kind of life lesson for them, that—at least on occasion—they might succumb too easily to pressure from others. The experimenters also answered any questions the participants had and encouraged them to make contact by phone or email (a special phone number and email address were provided) if they had any further questions or concerns. The entire debriefing procedure was performed by a clinical psychologist prepared for this role.

The research protocol was prepared in accordance with the Declaration of Helsinki. The full experimental procedure was discussed in advance with the SWPS University Ethics Committee and received its approval.

## Results

As a first step, we tested whether there were any gender differences in terms of obedience. Like Milgram and Berger, we found that none were present in either the sample as a whole or within individual conditions (χ^2^ < 1 in each of three comparisons). We thus did not consider the role of sex as a factor in further analyses.

**Figure 1:**
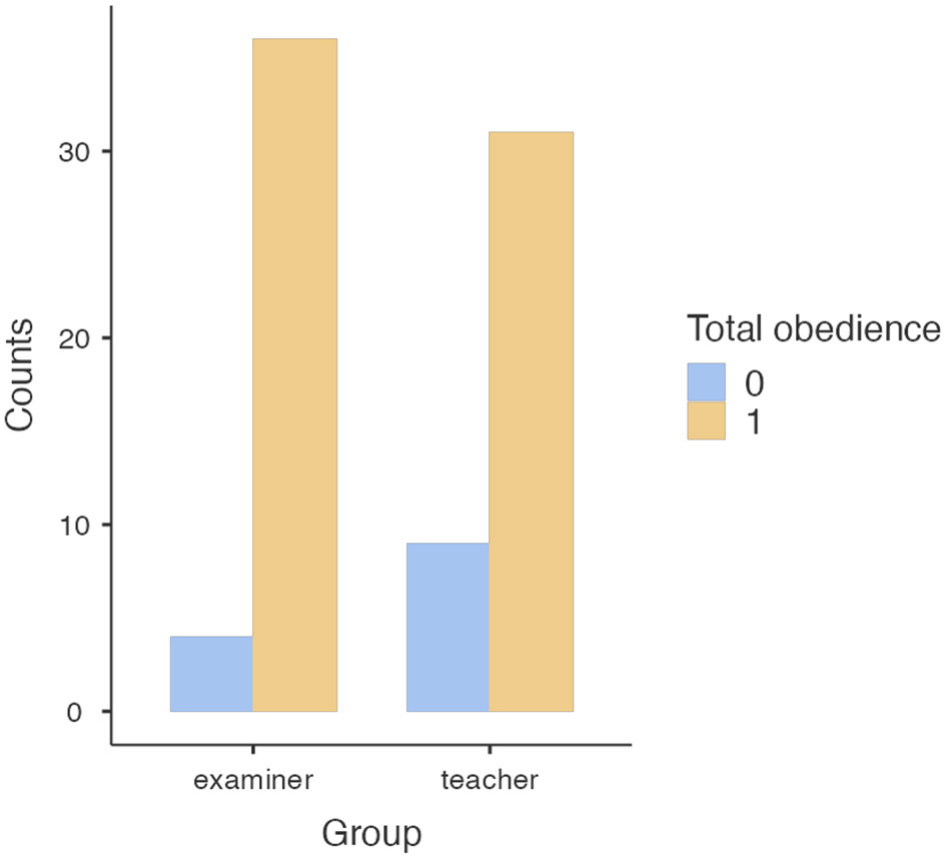
Number of participants refusing to obey in each experimental group.

In the examiner condition, 4 participants out of 40 (10%) refused to push all ten switches (the percentage of people who reached 150 V was 90%). In conditions in which the participant first taught the student, they refused more than twice as frequently: 9 out of 40 (22.5%) and the percentage of people who reached 150 V was 77.5%. However, this difference was not statistically significant: χ^2^_(1)_ = 2.30, *p* < 0.130, odds ratio = 2.61; 95% CI (0.73; 9.32). The detailed results are illustrated in Figure 1.

For a more precise examination of the relationship between the variables, a Bayesian analysis was conducted, since this method enabled a more precise evaluation of the strength of evidence. By relying on Bayes factors, it was possible to distinguish between data that actively supports the null hypothesis and data that merely fails to reject it, thereby providing a more informative interpretation of the observed effects. In both cases (for the quantitative variable—the last button pressed by the participants, and the dichotomous variable—full obedience or lack thereof), we used default priors with a zero-centered Cauchy distribution, *r* = 0.707. The result of the Bayesian Factor is BF_01_ = 2.337 for the quantitative variable and BF_01_ = 7.203 for the dichotomous variable. As can be observed, these results are not fully conclusive.

**Figure 2:**
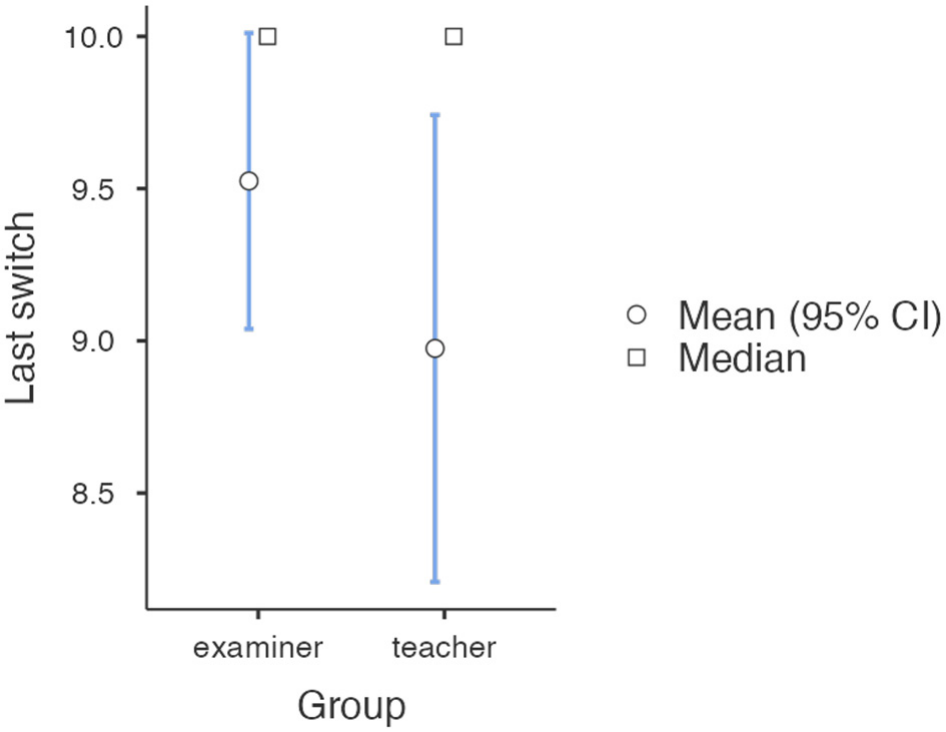
Mean number of prompts used in both experimental groups.

We also analyzed the frequency with which the experimenter had to motivate participants to press more switches, i.e., recite the phrases stipulated by the experimental procedure under conditions in which the participant expresses doubt or does not comply with the prods (“Please continue”, “The experiment requires that you continue”, “It is absolutely essential that you continue”, “You have no other choice, you must go on”). It turned out that such situationally enforced reactions by the experimenter were more frequent under conditions in which the participant had previously taught the student (Median = 0.5, Min = 0, Max = 4), than when acting as an examiner and only checking whether he had studied well student (Median=0, Min=0, Max=4). This difference proved to be statistically significant (*U Mann–Whitney*=596.5; *Z* = 2.241; *p* = 0.025, *eta*^2^ = 0.063). The detailed results are illustrated in Figure 2.

## Discussion

Although more than twice as many people refused to obey all of the experimenter's prods in the “teacher” condition than in the “examiner” condition, the difference did not attain statistical significance. An odds ratio of 2.61, however, suggests that there is a real difference in obedience to authority in the two conditions we created. What did prove statistically significant was the difference in the frequency of messages the experimenter had to utter to get participants to obey. Such situations were more frequent in conditions in which the participant first taught the student and only then reviewed his responses for correctness. Note that, for ethical reasons, the study was conducted in an obedience light procedure, ending when the participant pressed the tenth switch (or earlier, of course, when refusing further participation in the study). We note in this context that a precise analysis of participants' behavior in Milgram's experiments, in which they were prompted to press another 30 switches, showed that their expressions of doubt and hesitation before electrocuting the person sitting behind the wall is a good predictor of their later behaviors. Most often, participation in the experiment was terminated by those who previously had to be induced into action with phrases like “Please continue,” “The experiment requires that you continue,” etc. (Rochat and Blass, 2014).

Thus, it is reasonable to assume that our participants' expressions of doubt were also a predictor of their disobedience, which we did not observe simply because we ended our study much earlier than Milgram and other researchers did. However, even if we do not accept such an assumption, we should agree with the notion that the expression of verbal doubts or the absence of any response to the command to push the switch that triggers an electric shock indicates the presence of some intention for disobedience.

It seems, therefore, that the pattern of results we obtained indicates that one of the reasons why shockingly high levels of obedience are observed in experiments conducted in the Milgram paradigm is that the participants do not play the role of a teacher (which is erroneously assumed in many descriptions of this experiment), but rather the role of an examiner. Being an examiner is conducive to inducing an agentic state or social identification as a “good subject” and not feeling responsible for what happens to the student. The situation is different in conditions in which the examiner first teaches the student and only then tests his knowledge. In the first stage of this process, there is cooperation between the participant and the supposed student, which is an important factor that encourages the development of interpersonal bonds (Kuwabara, 2011). The results we obtained are consistent with the assumption that such a situation subsequently makes the participant feel more personally responsible for the student's mistakes, which, in turn, stops the participant from zapping him with electricity (or certainly at least raises doubts about whether it is right to do so).

Let's try to interpret our results through the lens of the engaged followership framework (see Birney et al., 2024, for a recent review). According to this account, individuals' willingness to harm others depends on their relative identification with the experimenter and the scientific enterprise. Conversely, identification with the learner as a fellow human being and community member should reduce participants' readiness to follow the experimenter's instructions. From this perspective, one might predict that participants who had been actively engaged in the scientific task from the outset—by teaching the learner—would be more inclined to obey and less prone to doubt its significance. On the other hand, it could equally be argued that participants who had previously taught the learner would also identify more strongly with him, in contrast to those whose role was limited to merely checking the correctness of his responses (i.e., acting as examiners). Thus, the concept of engaged followership does not yield a clear prediction as to whether participants assigned the role of a genuine teacher in Milgram's paradigm should display greater or lesser obedience compared to those instructed only to examine and punish errors.

Although the research we conducted concerns the determinants of obedience to authority, the most important conclusion that seems to emerge from the results we obtained pertains to the role that the teacher-student relationship plays in the educational process. In our study, just a few minutes spent by participants teaching another person tremendously influenced their subsequent behavior in the experiment. At a time when there is increasing talk of robots replacing humans in the teaching process (e.g., Fagin and Merkle, 2003; Barreto and Benitti, 2012), attention is most often paid solely to the efficiency of that process. Meanwhile, we would do well to remember that the role of a teacher is a broader one. Being a teacher is also about feeling responsibility for students and their conduct.

### Limitation and direction of future research

A critical reader might suggest an alternative interpretation of the pattern of results we observed. The differences between the two conditions in our experiment are not solely due to the participant being a teacher in one condition and merely an examiner in the other. Another potential factor is that the teacher spent more time with the student before the actual testing phase, compared to the examiner. Therefore, it could be argued that it was not the distinct roles the participants played, but rather the amount of time spent with the student during the initial phase of the experiment, that was crucial. However, this interpretation can be dismissed in light of other research conducted in the obedience-lite paradigm. In some studies, participants and confederates completed various tests and questionnaires together in a common room, maintaining eye contact and even conversing at times (Dolinski et al., 2017; Grzyb and Dolinski, 2023). These interactions had no effect on the participants' subsequent level of obedience.

However, it must be acknowledged that completely ruling out the alternative interpretation of our findings would require further research. Specifically, in addition to the two conditions we examined, it would be valuable to include conditions in which both individuals first engage in a joint activity (e.g., solving a crossword puzzle or assembling a jigsaw puzzle), after which one of them is ostensibly assigned the role of the learner and the other the role of the teacher, whose task is limited to checking the correctness of responses and administering penalties for mistakes.

Other promising directions for future research involve the inclusion of additional measures. To better understand the mechanisms underlying the finding that teachers are less likely than examiners to administer shocks, participants could be asked to what extent they felt responsible for the learner's performance, the degree of emotional attachment they experienced toward the learner, and whether they perceived the relationship with the learner in terms of “we.” Another valuable line of inquiry would involve incorporating psychophysiological and neuropsychological measures, in order to determine whether teachers and examiners differ on these dimensions as well.

When considering future research and theoretical analyses of obedience to authority, it is also important to examine how our results relate to existing conceptual frameworks. For instance, in the model proposed by Russell and Künstler (2024), prior preparation of the learner for the task may increase participants' motivation to overcome resistance to direct confrontation with the experimenter. The relationship between our findings and the engaged followership account, however, is more complex. Notably, the teacher role appears to increase identification with *science*, but not with the *experimenter*. Haslam and Reicher treat these (i.e., identification with science and with experimenter) as a single construct, but we argue that this is a mistaken conflation. Finally, our result—reduced obedience in the teacher condition compared to the examiner condition—is fully consistent with our own assumption (Dolinski and Grzyb, 2024, 2025) that participants in Milgram's paradigm experience an avoidance–avoidance conflict: they do not want to harm the learner, yet they also do not wish to refuse the experimenter and disappoint his expectations. Assigning the role of teacher heightens the aversiveness of harming the learner, thereby altering the way this conflict is experienced and ultimately resolved, compared to conditions in which the participant functions as an examiner.

## Data Availability

The datasets and materials generated during the current study are available via this link https://osf.io/dbymr/?view_only=f87f9a3435e441fba279b61ca982ee42. Further inquiries can be directed to TG.
